# Anisotropic Polymer Adsorption on Molybdenite Basal and Edge Surfaces and Interaction Mechanism With Air Bubbles

**DOI:** 10.3389/fchem.2018.00361

**Published:** 2018-08-20

**Authors:** Lei Xie, Jingyi Wang, Jun Huang, Xin Cui, Xiaogang Wang, Qingxia Liu, Hao Zhang, Qi Liu, Hongbo Zeng

**Affiliations:** ^1^Department of Chemical and Materials Engineering, University of Alberta, Edmonton, AB, Canada; ^2^College of Material Science and Engineering, Heavy Machinery Engineering Research Center of Education Ministry, Taiyuan University of Science and Technology, Taiyuan, China

**Keywords:** polymer depressant, molybdenite (MoS_2_) basal/edge, anisotropic adsorption, froth flotation, atomic force microscope (AFM), adhesion, bubble probe AFM

## Abstract

The anisotropic surface characteristics and interaction mechanisms of molybdenite (MoS_2_) basal and edge planes have attracted much research interest in many interfacial processes such as froth flotation. In this work, the adsorption of a polymer depressant [i.e., carboxymethyl cellulose (CMC)] on both MoS_2_ basal and edge surfaces as well as their interaction mechanisms with air bubbles have been characterized by atomic force microscope (AFM) imaging and quantitative force measurements. AFM imaging showed that the polymer coverage on the basal plane increased with elevating polymer concentration, with the formation of a compact polymer layer at 100 ppm CMC; however, the polymer adsorption was much weaker on the edge plane. The anisotropy in polymer adsorption on MoS_2_ basal and edge surfaces coincided with water contact angle results. Direct force measurements using CMC functionalized AFM tips revealed that the adhesion on the basal plane was about an order of magnitude higher than that on the edge plane, supporting the anisotropic CMC adsorption behaviors. Such adhesion difference could be attributed to their difference in surface hydrophobicity and surface charge, with weakened hydrophobic attraction and strengthened electrostatic repulsion between the polymers and edge plane. Force measurements using a bubble probe AFM showed that air bubble could attach to the basal plane during approach, which could be effectively inhibited after polymer adsorption. The edge surface, due to the negligible polymer adsorption, showed similar interaction behaviors with air bubbles before and after polymer treatment. This work provides useful information on the adsorption of polymers on MoS_2_ basal/edge surfaces as well as their interaction mechanism with air bubbles at the nanoscale, with implications for the design and development of effective polymer additives to mediate the bubble attachment on solid particles with anisotropic surface properties in mineral flotation and other engineering processes.

## Introduction

Molybdenite (MoS_2_), the most important mineral source of molybdenum, has exhibited excellent characteristics and functionality in a broad range of biomedical and engineering applications, such as hydrogen evolution catalysis (Jaramillo et al., 2007; Karunadasa et al., 2012; Li et al., 2016; Liu et al., 2016), friction and lubrication (Chhowalla and Amaratunga, 2000; Lee et al., 2010; Sheehan and Lieber, 2017), and clinical devices (Liu et al., 2014; Yin et al., 2014). The consecutive S-Mo-S layers held by weak van der Waals (vdW) interaction can be readily exfoliated, generating the basal plane that is inherently hydrophobic in nature with ultra-low friction (Chhowalla and Amaratunga, 2000; Lee et al., 2010; Sheehan and Lieber, 2017). On the other hand, MoS_2_ edge generated by breaking Mo-S covalent bonds is relatively hydrophilic, with great potential as an alternative electrocatalyst for hydrogen evolution reaction (Jaramillo et al., 2007; Karunadasa et al., 2012; Li et al., 2016). The anisotropic surface properties of MoS_2_ basal and edge planes can significantly influence their interaction behaviors at air-water-solid interfaces. In froth flotation, MoS_2_ minerals are selectively separated from other mineral particles (e.g., chalcopyrite, talc). The basal to edge ratio of MoS_2_ minerals greatly impacts their flotation performance, depending on the attachment propensity of air bubbles to both the basal and edge planes (Fuerstenau et al., 2007; Rao, 2013). Therefore, understanding the anisotropic surface properties and interaction mechanisms of MoS_2_ basal and edge planes with air bubbles is of both fundamental and practical importance.

Over the last several decades, the anisotropy in MoS_2_ basal and edge surfaces has attracted much research interest (Okuhara and Tanaka, 1978; Roxlo et al., 1986; Zhang et al., 2004; Gaur et al., 2014; Jin et al., 2014; Lu et al., 2015; Tan et al., 2015; Govind Rajan et al., 2016; Bentley et al., 2017). The molecular dynamics simulations showed the water contact angle of 54° for the armchair-edge and 24° for the zigzag-edge of MoS_2_, much lower than that of 83° for MoS_2_ basal plane (Jin et al., 2014). The edge plane was found to exhibit more negative surface potential than the basal plane in neutral and alkaline pH conditions, and the hydrophobic interaction was only detected on the basal plane (Lu et al., 2015). In molybdenum-containing ores, water-soluble polymers are widely used as the depressants to alter the surface characteristics of MoS_2_ basal planes (Pugh, 1989; Liu et al., 2000; Pearse, 2005; Bulatovic, 2007; Beaussart et al., 2009; Kor et al., 2014; Castro et al., 2016). The adsorption of polymer depressants [e.g., dextrin, carboxymethyl cellulose (CMC)] on MoS_2_ basal plane was reported to depend on the solution condition and polymer charge density. The adsorption of these polymers could lead to a reduced surface hydrophobicity and slower rate of dewetting process during the single bubble collision study (Beaussart et al., 2009; Kor et al., 2014). To date, no report is available to distinguish the adsorption mechanisms of polymer depressants on the MoS_2_ basal planes from the MoS_2_ edge planes, nor are there quantitative measurements of their interaction forces with air bubbles.

Atomic force microscope (AFM) and surface forces apparatus (SFA) have been widely employed to measure the surface forces and interaction mechanisms of various solid material systems in vapor and liquid media at the nanoscale (Ducker et al., 1991; Butt et al., 2005; Israelachvili et al., 2010; Greene et al., 2011; Huang et al., 2014; Kristiansen et al., 2014; Yang et al., 2014; Wang et al., 2016, 2017; Xie et al., 2016, 2017c; Zhang et al., 2016; Gao et al., 2018). The force-distance profiles and the adhesion magnitude are closely correlated to a variety of interfacial phenomena, including adsorption, aggregation and deposition. The adsorption of protein on polymer surfaces has been investigated by measuring the interaction and adhesion between the protein coated probe and polymer surfaces (Chen et al., 1997; Taylor et al., 2008). Recently, a polydopamine (PDA) coated AFM probe has been employed to investigate the interaction between PDA and solid surfaces of varied hydrophobicity, aiming to understand the adsorption, deposition and adhesion of PDA on solids (Zhang et al., 2017). Thus, force measurements using a polymer coated probe could be used to study the interaction of polymers with mineral surfaces, which is highly relevant to the polymer adsorption on minerals. The limitation of force measurements using the polymer coated probe is that the polymers adsorbed on the AFM probe would perform differently from polymers in aqueous solution. Apparently, the exploit of polymer coated AFM probe can provide the comparable information on the polymer adsorption on different surfaces, but the adsorption capability of polymers cannot be directly and quantitatively provided. The bubble/drop probe AFM technique has also been developed and applied to study the interaction mechanisms involving deformable gas bubbles and emulsion drops, enabling the precise quantification of surface forces at air/water and oil/water interfaces with sub-nN resolution (Manor et al., 2008; Chan et al., 2011; Tabor et al., 2011; Shi et al., 2014a,b, 2016a,b, 2017; Xie et al., 2015, 2017a,b,d; Cui et al., 2016, 2017; Rocha et al., 2016). Very recently, the effect of aqueous conditions (e.g., pH, salinity and salts) on the interaction forces between air bubble and bitumen surface was quantitatively measured, and the evolution of confined thin water film drainage process was successfully analyzed using the theoretical model based on the Reynolds lubrication theory and augmented Young-Laplace equation (Xie et al., 2017d). It was found that the complex aqueous media effectively affected the bubble-surface attachment behaviors by mediating the electrical double layer (EDL) repulsion and hydrophobic attraction (Xie et al., 2017a). The bubble probe AFM technique coupled with other complementary surface analytic tools enables the investigation of the anisotropic adsorption and associated interaction mechanisms on MoS_2_ basal and edge planes.

In this work, the thin edge plane of MoS_2_ with relatively large surface area (15 × 5 mm^2^) and low root-mean-square (rms) roughness (~1.6 nm) was prepared by carefully polishing the edge sample (see Materials and Methods) (Wang et al., 2013), which could be used for AFM imaging and force measurements in a fluid cell. The effect of adsorbed polymer (i.e., CMC) on the wettability and morphology of MoS_2_ basal and edge surfaces was compared using contact angle measurement and *in-situ* AFM imaging. The adsorption mechanism of polymer depressants was investigated by measuring their intermolecular forces and adhesion on both MoS_2_ basal and edge surfaces using CMC functionalized AFM tips. In addition, the bubble probe AFM technique was employed to quantitatively measure the interaction forces between an air bubble and MoS_2_ basal or edge surfaces in aqueous solution with or without the influence of polymer adsorption. This work provides valuable information regarding the polymer adsorption and bubble interaction mechanism on the anisotropic basal and edge planes of MoS_2_ at the nanoscale. The results show useful implications for the design and development of effective polymer additives to mediate the bubble attachment on solid particles with anisotropic surface properties in froth flotation and many other interfacial processes.

## Materials and methods

### Materials

Sodium chloride (NaCl, ACS reagent grade), hydrochloric acid (HCl, ACS reagent grade) and sodium hydroxide (NaOH, ACS reagent grade) were purchased from Fisher Scientific Canada and used as received without further purification. (3-Aminopropyl)triethoxysilane (APTES) and sodium carboxymethyl cellulose (CMC, molecular weight M_W_ ~2.5 × 10^5^ g/mol, degree of substitution DS ~1.2) were purchased from Sigma Aldrich. All aqueous solutions were prepared using Milli-Q water (Millipore deionized, 18.2 MΩ·cm resistivity), and the pH was adjusted and fixed at 9 in this work.

### Preparation of MoS_2_ basal and edge planes

**Figure 1:**
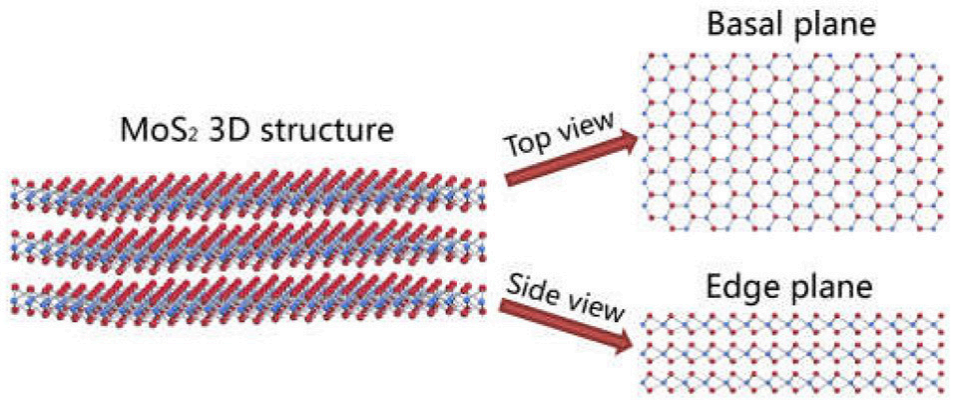
Illustration of MoS_2_ crystal structure: 3D structure, top view (basal plane) and side view (edge plane) (Color red: sulfur atom and color blue: molybdenum atom).

The MoS_2_ bulk minerals (Ward's Science, Rochester, NY) were embedded in epoxy resin for preparing the edge surface. After curing overnight, the epoxy samples were polished by hand with wet silicon carbide paper of 60 grit to expose the edge plane. The smooth edge surface was obtained by polishing with wet silicon carbide paper of 60, 240, 320, 400, 600, 800, and 1200 grit, and then polishing with 5, 1, and 0.3 μm alumina powder suspension, respectively (Wang et al., 2013). The freshly polished edge samples were ultrasonically washed in Milli-Q water, ethanol and Milli-Q water for 5 min, respectively. The natural cleavage surface of the basal plane (1 × 1 cm^2^) was obtained by peeling off the top layers using a sticky tape. Specifically, the thin MoS_2_ sample was fixed on a glass slide using double sided adhesive tape, and Scotch tape was then applied to remove the top layers of MoS_2_ sample. After repeating the peeling for over three times to avoid the contamination, the prepared basal plane was observed using the microscopy until a desired surface was detected. The freshly prepared MoS_2_ basal and edge surfaces were immediately used for surface characterizations and polymer adsorption tests. Figure 1 shows the illustration of the 3D structure, top view (basal plane) and side view (edge plane) of a typical MoS_2_ crystal.

The polymer stock solution (~100 ppm) was prepared by dissolving a desired amount of CMC in Milli-Q water under stirring overnight to ensure complete hydration. By diluting the stock solution in Milli-Q water and adjusting the pH to 9, solutions of desired concentration (i.e., 5 and 10 ppm) were obtained. Thereafter, the freshly prepared MoS_2_ basal and edge planes were conditioned in the desired polymer solutions for further surface characterization.

### Surface characterization

A contact angle goniometer (ramé-hart instrument Co., NJ, USA) was used to measure the static water contact angle on MoS_2_ basal and edge planes using a sessile drop method. For the same type of sample, at least two different surfaces and three different locations on each surface were tested, and the average water contact angle was reported. The topographic images of MoS_2_ basal and edge planes were obtained using the tapping mode of an AFM.

### Adsorption mechanism of CMC

**Figure 2:**
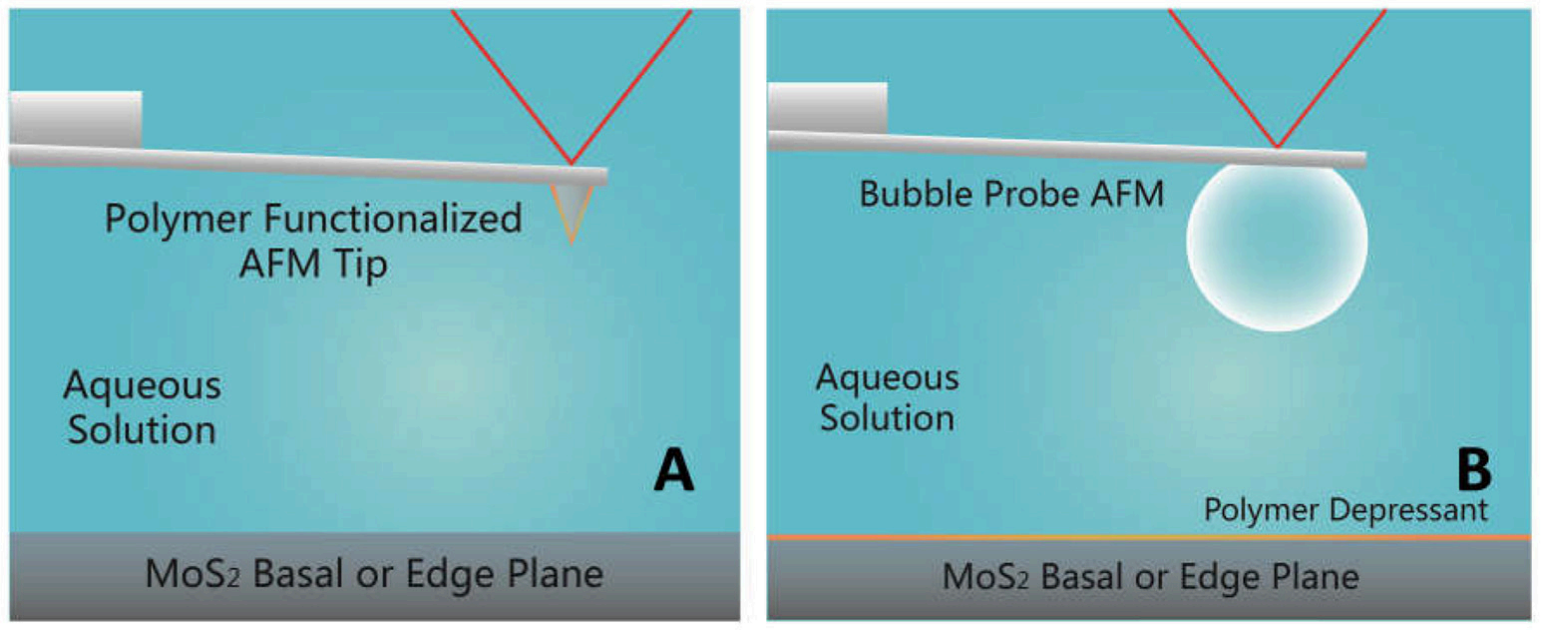
Schematic of force measurements **(A)** between polymer functionalized AFM tip and MoS_2_ basal or edge plane, and **(B)** between air bubble and MoS_2_ basal or edge plane using the bubble probe AFM.

The interaction between a CMC functionalized AFM tip and a MoS_2_ basal or edge plane was measured in 1 mM NaCl at pH 9 using a MFP-3D AFM (Asylum Research, Santa Barbara, CA, USA). Prior to force measurements, AFM silicon probes were cleaned by UV/ozone treatment for 30 min and coated with APTES through a vapor deposition process for 4 h (Lu et al., 2011). The APTES coated AFM probes were immersed in 100 ppm CMC solution overnight, after which the prepared AFM probes were washed with Milli-Q water and positioned over the mineral surface for force measurements. The spring constant of the probe was determined to be 0.1–0.2 N/m using the Hutter and Bechhoefer method (Hutter and Bechhoefer, 1993). The schematic of typical experiment setup for measuring the interaction forces between the CMC functionalized AFM tip and MoS_2_ surface is shown in Figure 2A.

### Bubble-MoS_2_ interaction

The interaction between an air bubble and a MoS_2_ basal or edge plane was measured in 500 mM NaCl at pH 9 using the bubble probe AFM. Prior to force measurements, the glass disk of a fluid cell was mildly hydrophobized by immersing in 10 mM octadecyltrichlorosilane (OTS) in toluene for ~10 s for bubble immobilization; while custom-made rectangular AFM cantilevers (400 × 70 × 2 μm) with a circular gold patch (diameter ~65 μm, thickness ~30 nm) were strongly hydrophobized by immersing in 10 mM dodecanethiol in absolute ethanol overnight to provide higher hydrophobicity than the glass disk for bubble anchoring (Shi et al., 2014b). Air bubbles were generated and immobilized on the glass disk by carefully purging air through a custom-made ultra-sharp glass pipette into the aqueous solution. The bubble probe was then prepared by picking up an air bubble of suitable size (typically 50–90 μm radius) with the AFM cantilever. The cantilever-anchored air bubble was positioned over the mineral surface and then driven to approach the substrate surface until a fixed deflection of the cantilever was reached or bubble attachment occurred. The spring constant of the cantilever was determined to be 0.3–0.4 N/m (Hutter and Bechhoefer, 1993). The force measurements were performed at a driving velocity of 1 μm/s, under which the effect of hydrodynamic interaction was negligible as compared to surface forces. The movement of the cantilever (anchored with air bubble) and the corresponding interaction forces were recorded as a function of time by AFM software. The schematic of typical experiment setup for force measurements on MoS_2_ surface using the bubble probe AFM is shown in Figure 2B.

### Theoretical model

A theoretical model based on the Reynolds lubrication theory coupled with augmented Young-Laplace equation was applied to analyze the experimentally measured forces between air bubbles and mineral surfaces.

The bubble deformation under the combined influence of hydrodynamic interaction and disjoining pressure is given by the augmented Young-Laplace equation (Shi et al., 2014a,b; Xie et al., 2015).

(1) $$\begin{array}{r} \frac{\gamma }{r}\frac{\partial }{\partial r}\left(r\frac{\partial h}{\partial r}\right)=\frac{2\gamma }{R}-p-\prod \end{array}$$

where γ is the surface tension of water, *h*(*r, t*) is the thickness of confined thin water film, *R* is the bubble radius, *p*(*r, t*) is the excess hydrodynamic pressure in the water film relative to the bulk solution, and Π(*r, t*) is the overall disjoining pressure arising from surface forces such as vdW, EDL, and hydrophobic interactions.

The Reynolds lubrication theory describes the drainage process of thin water films confined between air bubbles and mineral surfaces (Shi et al., 2014a,b; Xie et al., 2015).

(2) $$\begin{array}{r} \frac{\partial h}{\partial t}=\frac{1}{12\mu r}\frac{\partial }{\partial r}\left(rh^{3}\frac{\partial p}{\partial r}\right) \end{array}$$

where μ is the dynamic viscosity of water. Consistent with recent reports, immobile boundary condition was assumed at air-water and water-mineral interfaces (Shi et al., 2014a,b; Xie et al., 2015).

In this work, high salinity condition (i.e., 500 mM NaCl) was chosen to suppress the EDL interaction, with a Debye length less than 1 nm. The contributions of vdW and hydrophobic interactions to the disjoining pressure Π_*vdW*_ and Π_*HB*_ are described by equations 3 and 4, respectively, where *A* is the Hamaker constant for air-water-MoS_2_ (*A* = −2.68 × 10^−20^ J), *D*_0_ is the decay length of hydrophobic interaction and *C* is a constant (N/m) related to the static water contact angle θ on the substrate and surface tension of water γ (Israelachvili, 2011; Xie et al., 2015).

(3) $$\begin{array}{rcl} \prod_{vdW} & = & -\frac{A}{6\pi h^{3}} \end{array}$$

(4) $$\begin{array}{rcl} \prod_{HB} & = & -\frac{C}{2\pi D_{0}}e^{-h/D_{0}}=-\frac{\gamma \left(1-\cos\theta \right)}{D_{0}}e^{-h/D_{0}} \end{array}$$

The overall interaction force *F*(*t*) between an air bubble and a mineral surface is theoretically calculated by integrating *p*(*r, t*) and Π(*r, t*) based on the Derjaguin approximation in equation 5 (Shi et al., 2014a,b; Xie et al., 2015).

(5) $$\begin{array}{r} F\left(t\right)=2\pi \overset{\infty }{\underset{0}{\int }}\left(p\left(r,t\right)+\prod \left(h\left(r,t\right)\right)\right)rdr \end{array}$$

## Results and discussion

### Surface morphology

**Figure 3:**
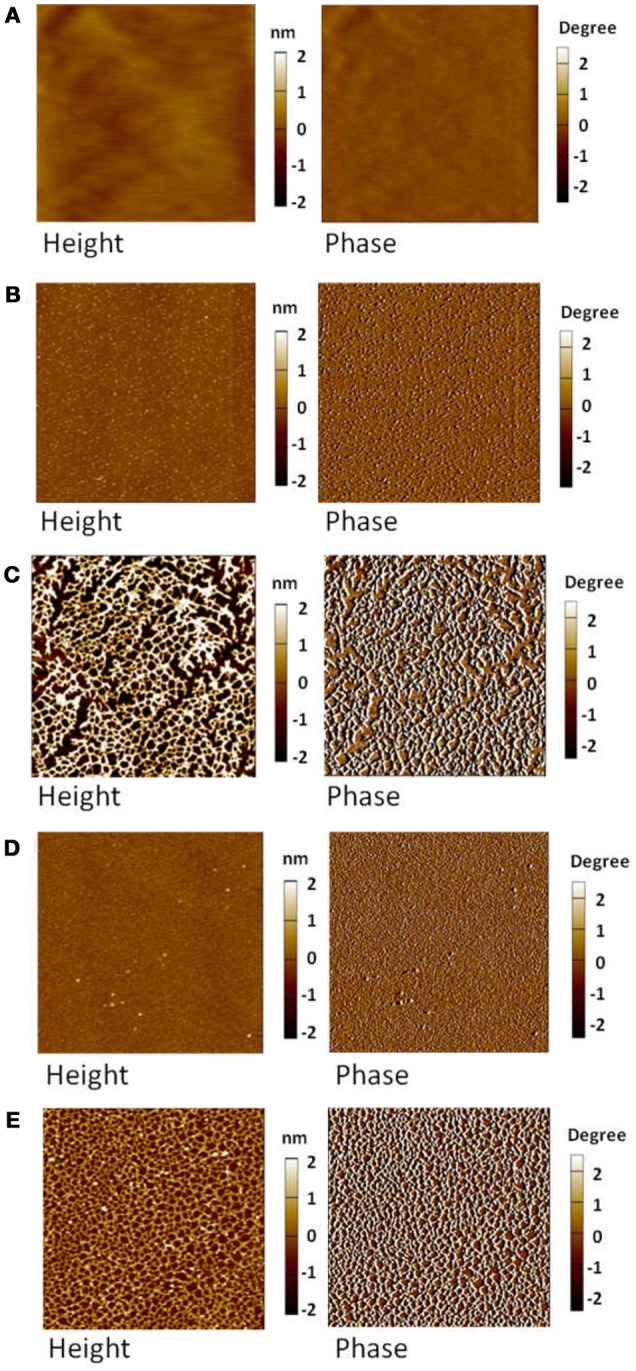
AFM height (**Left**) and phase (**Right**) images of MoS_2_ basal plane conditioned at different CMC concentrations in Milli-Q water at pH 9 for 30 min: **(A)** 0 ppm (5 × 5 μm^2^), **(B)** 5 ppm (5 × 5 μm^2^), **(C)** 10 ppm (5 × 5 μm^2^), **(D)** 100 ppm (5 × 5 μm^2^), and **(E)** 100 ppm (2 × 2 μm^2^).

Figure 3 shows the AFM images of MoS_2_ basal planes conditioned at different CMC concentrations at pH 9 for 30 min. Figure 3A demonstrated that the basal plane generated by exfoliation along the vdW gap is molecularly smooth with the rms roughness of ~0.20 nm, which is consistent with our previous result (Xie et al., 2017d). After polymer adsorption for 30 min, evident variations in the morphology of the basal planes (5 × 5 μm^2^) conditioned in CMC solutions of different concentrations could be detected from both the height and phase images in Figures 3B–D. The basal plane conditioned in 5 ppm CMC solution in Figure 3B showed randomly and sparsely distributed aggregates (bright spots) that exhibited apparent phase difference with surrounding areas. Thus, the formed aggregates were attributed to the adsorbed polymers, which covered ~14.8% of the basal plane. With CMC concentration increasing to 10 ppm, an interconnected polymer network formed on the basal plane with the surface coverage achieving ~47.1% in Figure 3C. After conditioned in 100 ppm CMC solution, Figure 3D showed the formation of a smooth polymer film on the basal plane. The magnified image (2 × 2 μm^2^) of the polymer film formed in 100 ppm CMC solution showed the interconnected polymer network (Figure 3E), which was more closely compact than the 10 ppm case in Figure 3C, indicating that the full polymer coverage was not be achieved even at the CMC concentration as high as 100 ppm. Overall, the polymer concentration plays a significant role in the polymer adsorption on MoS_2_ basal plane. In our previous study, the adsorption of guar gum on MoS_2_ basal plane was found to lead to the formation of interconnected polymer network at 5 ppm and a fully covered polymer film at 10 ppm (Xie et al., 2017d). It is evident that the adsorption of CMC is more difficult than guar gum most likely due to the negatively charged carboxyl groups of CMC that could induce stronger electrostatic repulsion with the negatively charged MoS_2_ basal plane (surface potential at pH 9: −55 mV in 1 mM NaCl and −44 mV in 10 mM NaCl) (Lu et al., 2015; Xie et al., 2017d).

**Figure 4:**
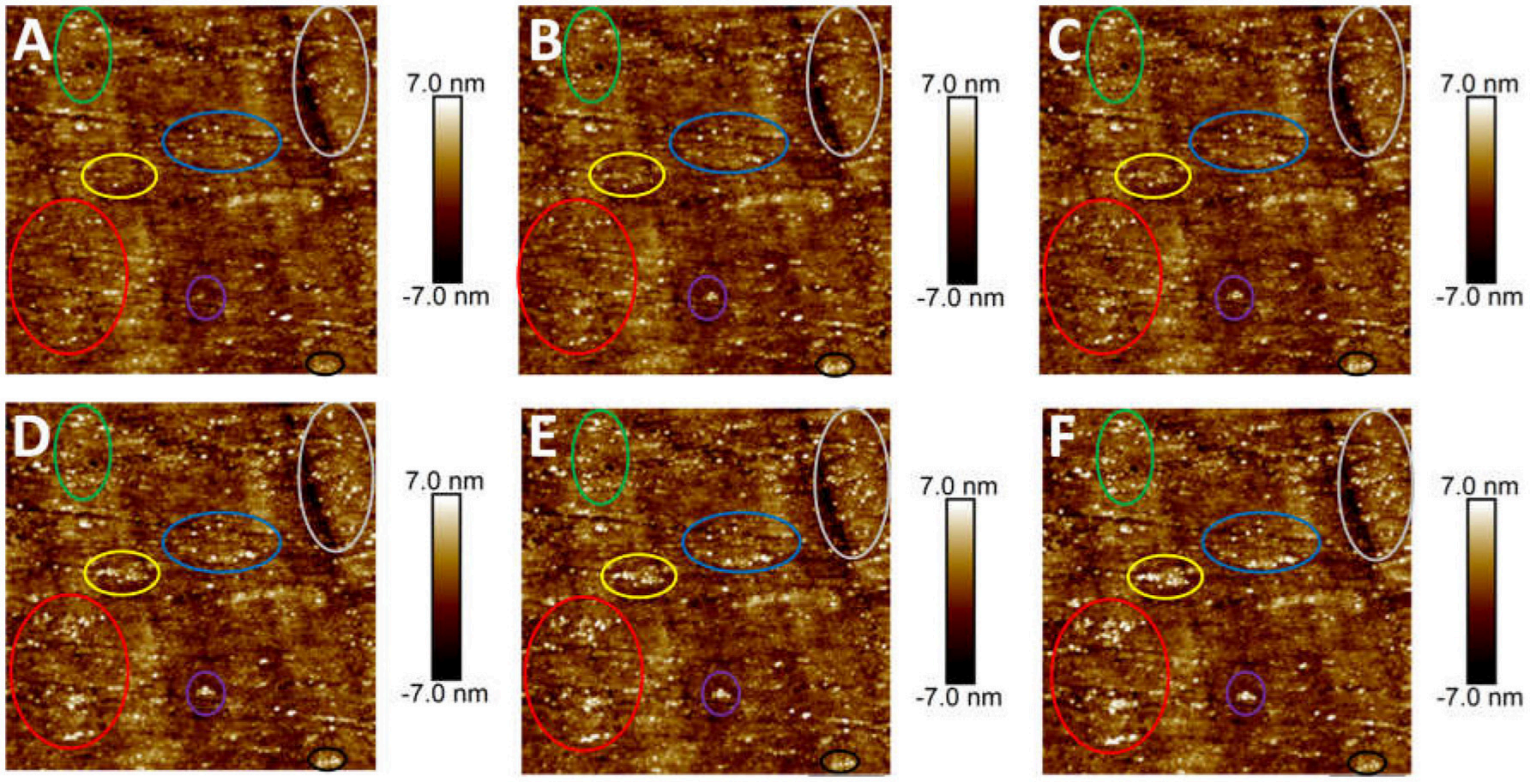
AFM height images (5 × 5 μm^2^) of MoS_2_ edge plane conditioned in 100 ppm CMC solution in Milli-Q water at pH 9 for different adsorption times: **(A)** 0 min, **(B)** 30 min, **(C)** 60 min, **(D)** 90 min, **(E)** 120 min, and **(F)** 180 min.

The adsorption of CMC on MoS_2_ edge plane was also investigated using AFM imaging. On the topographic AFM image of freshly polished edge plane shown in Figure 4A, the dark regions were polishing defects that could not be totally avoided, while the bright regions were the aggregates possibly arising from the edge crystal structure. The rms roughness of the polished edge plane was measured to be 1.61 nm, which was smoother than the MoS_2_ edge plane obtained by the ultramicrotome cutting technique (1.6–3.3 nm) reported previously (Lu et al., 2015, 2016). Since the edge plane was not as molecularly smooth as the basal plane, the polishing defects and aggregates pre-existed on the edge plane might affect the determination of adsorbed polymer. To better monitor the evolution of polymer adsorption on the edge plane, Figure 4 shows the *in-situ* topographic AFM images scanned over the same 5 × 5 μm^2^ region in 100 ppm CMC solution at pH 9. Figures 4B–F showed that slight variation in the surface morphology could be observed during the polymer treatment at different times. Certain aggregates were detected after polymer adsorption and the size of aggregation domains (circled in different colors) increased with longer adsorption time. As the adsorption time increased from 30 min to 60, 90, 120, and 180 min, the rms roughness increased from 1.70 nm to 1.78, 1.85, 1.86, and 1.91 nm, respectively. Both the *in-situ* evolution of aggregation domains and increased rms roughness obtained from AFM imaging indicate that CMC can only slightly adsorb on MoS_2_ edge surface, although it would be difficult to differentiate if CMC might preferentially adsorb on the polishing defects. As compared to the formation of a smooth polymer film on the basal plane (Figure 3D), the polymer adsorption on the edge plane (Figure 4B) at the same polymer concentration (i.e., 100 pm) for the same adsorption time (i.e., 30 min) seemed almost negligible.

### Surface wettability

**Figure 5:**
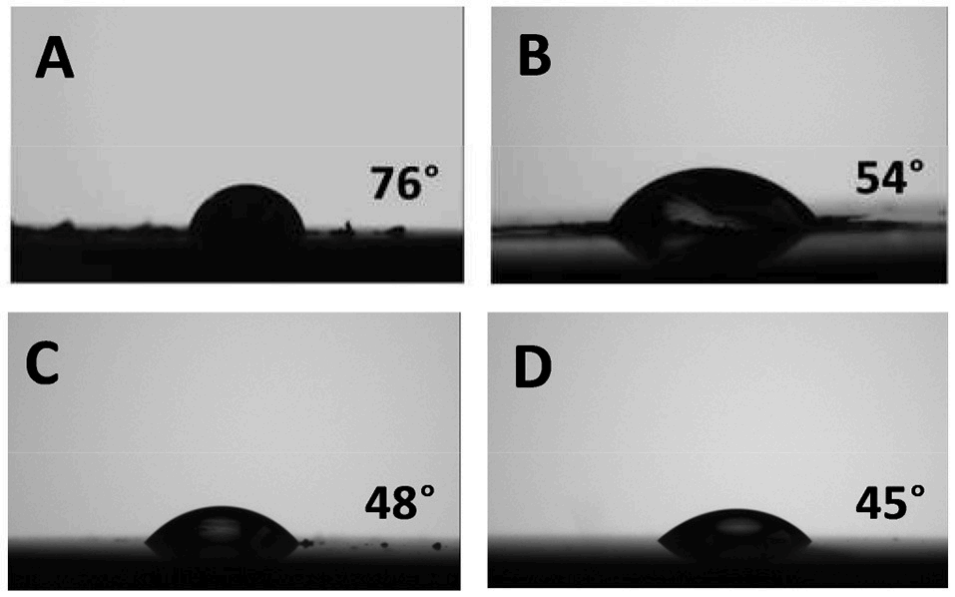
Water contact angle of MoS_2_ basal plane **(A)** before and **(B)** after polymer adsorption, and MoS_2_ edge plane **(C)** before and **(D)** after polymer adsorption, in 100 ppm CMC solution at pH 9 for 30 min.

Figure 5 shows the typical microscope images of water contact angle on MoS_2_ basal and edge surfaces before and after conditioning in 100 ppm CMC solution at pH 9 for 30 min. The freshly exfoliated basal plane exhibited inherent hydrophobicity with a static water contact angle of 76° (Figure 5A), which decreased to 54° after polymer adsorption (Figure 5B), indicating that CMC is an efficient polymer depressant to reduce the hydrophobicity of MoS_2_ basal surface. On the other hand, the freshly polished edge plane is relatively hydrophilic with a static water contact angle of 48° (Figure 5C), and the water contact angle almost remained unchanged after polymer adsorption (Figure 5D), suggesting the negligible CMC adsorption on MoS_2_ edge surface. The anisotropy in water contact angle of MoS_2_ basal and edge planes agreed well with AFM imaging results on the effect of polymer adsorption (Figures 3, 4).

### Adsorption mechanism of CMC

**Figure 6:**
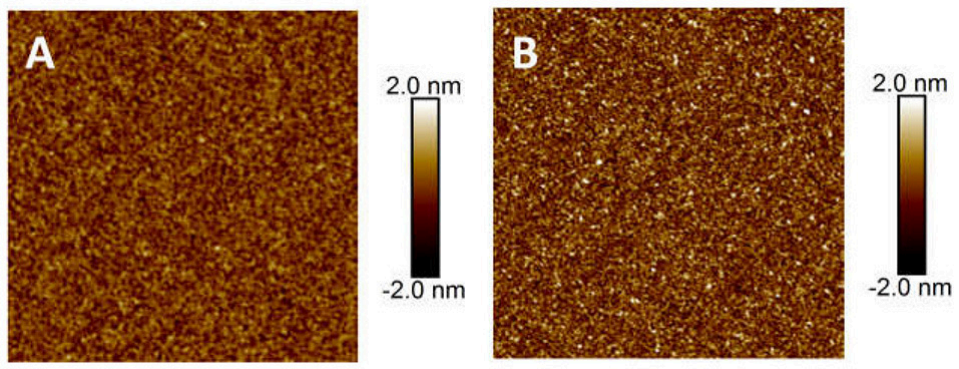
AFM height images (5 × 5 μm^2^) of **(A)** APTES coated silicon and **(B)** adsorbed CMC on APTES coating.

To investigate the adsorption mechanism of CMC on MoS_2_ basal and edge surfaces, the CMC functionalized AFM tip was used to measure the interaction with the basal and edge surfaces. The CMC functionalized AFM tips were prepared by coating AFM silicon probes with APTES that enabled the adsorption of CMC through both chemical and physical interactions (e.g., amide bond, electrostatic attraction, hydrophobic attraction). The APTES coating and the follow-up CMC coating were examined by measuring the water contact angle and imaging the topography on silicon wafers that were treated using the same procedure as AFM silicon probes. The fresh silicon wafer, APTES coating and CMC coating showed the water contact angle of 9.5°, 58.4°, and 33.1°, respectively, indicating the successful coating of APTES on the silicon wafer and the adsorption of CMC on the APTES coating. Figure 6 shows the AFM images of APTES coating and CMC coating. The prepared APTES coating was molecularly smooth with a rms roughness of ~0.34 nm. After polymer adsorption overnight, the surface became rougher and the measured rms roughness increased to ~0.54 nm. The difference in surface morphology further demonstrated the successful and uniform adsorption of CMC on APTES coating.

**Figure 7:**
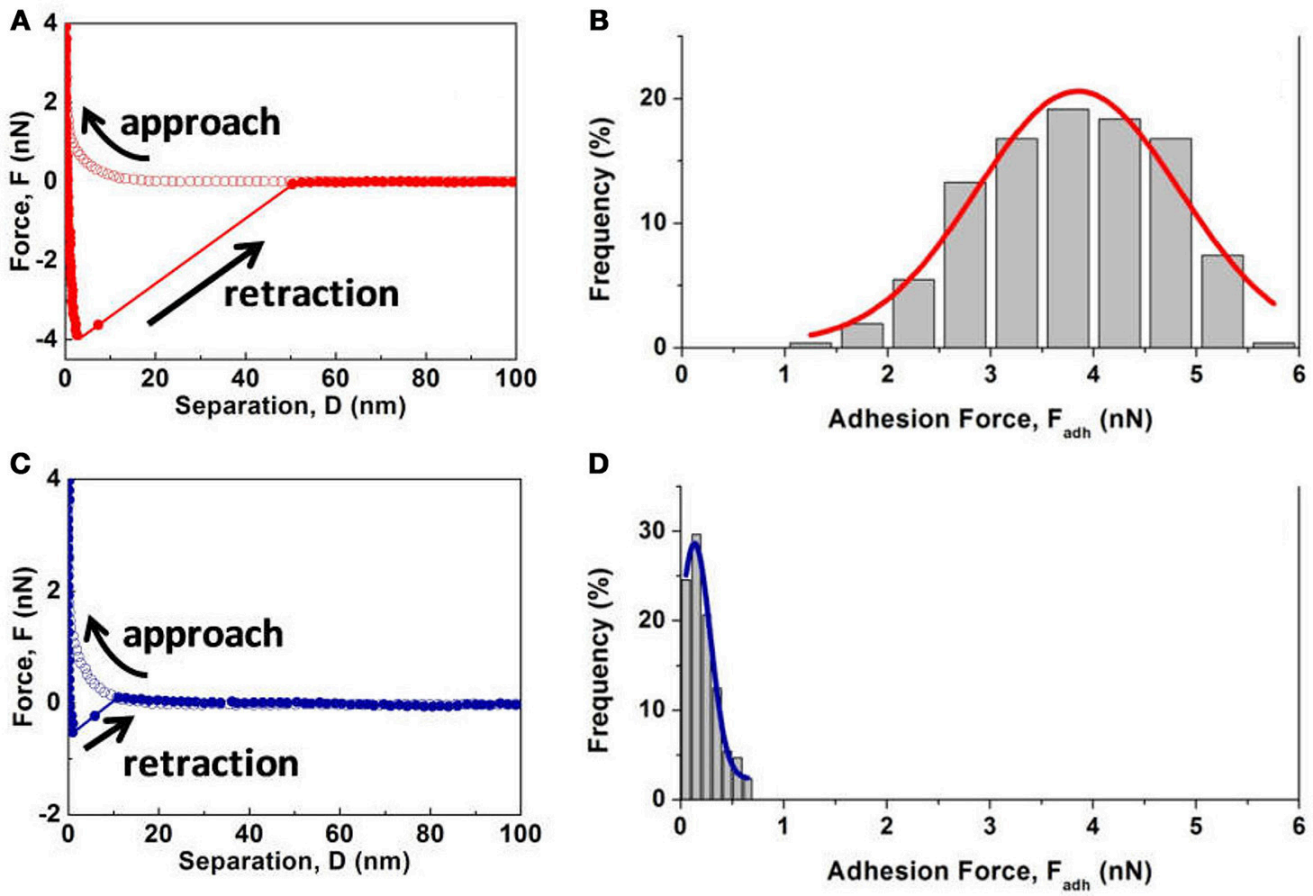
**(A,C)** Typical force-separation curves and **(B,D)** histograms of measured adhesion *F*_*adh*_ between CMC functionalized AFM tip and **(A,B)** MoS_2_ basal plane or **(C,D)** edge plane in 1 mM NaCl at pH 9.

Figure 7 shows the interaction forces measured between CMC functionalized AFM tip and MoS_2_ basal or edge plane in 1 mM NaCl at pH 9. The typical force-separation curves in Figures 7A,C show very strong repulsion during approach for both the basal and edge surfaces, attributed to the EDL repulsion and steric repulsion between the negatively charged CMC and MoS_2_. When the CMC functionalized AFM tip was retracted from the basal plane (Figure 7A), a strong jump-out behavior was detected, indicating strong adhesion between CMC and the basal plane. In contrast, as illustrated in Figure 7C, the adhesion on the edge plane was about one order of magnitude weaker. The histograms of measured adhesion *F*_*adh*_ and the fitted Gaussian distribution (red curve) in Figures 7C,D show that the adhesion distribution on the basal plane falls in a range of 1.0 to 6.0 nN with the fitted peak centered at 3.85 nN, much larger than that on the edge plane (centered at 0.13 nN), which supported the anisotropic adsorption of CMC on MoS_2_ basal and edge planes in Figures 3, 4. The observed adhesion might be originated from hydrogen bonding and other interactions (e.g., vdW and hydrophobic interactions). The exposed layer of MoS_2_ basal plane is composed of S atoms, while the polished edge plane is composed of both Mo and S atoms. The negligible polymer adsorption and very weak adhesion on the edge plane suggest that the formation of either chemical bond (with S or Mo atom) or hydrogen bond (with S atom) is not the main cause of CMC adsorption on MoS_2_. Since the vdW interaction between polymer and basal or edge surface is similar due to the same MoS_2_ bulk composition, the polymer adsorption would be most likely due to the hydrophobic attraction between the mineral surface and hydrophobic moieties of polymer chains. Such adhesion difference on the basal and edge surfaces could be attributed to their difference in surface hydrophobicity (water contact angle: 76° for the basal and 54° for the edge) and surface charge (surface potential in 10 mM NaCl at pH 9: −44 mV for the basal and −69 mV for the edge) (Lu et al., 2015), with weakened hydrophobic attraction and strengthened electrostatic repulsion between the polymer and edge plane leading to much weaker adhesion. Ab-initio modeling can provide useful information on the interaction between CMC and MoS_2_ basal/edge surface to supplement the force measurements in this work, which will be further studied and reported in a separate work.

### Bubble-MoS_2_ interaction

**Figure 8:**
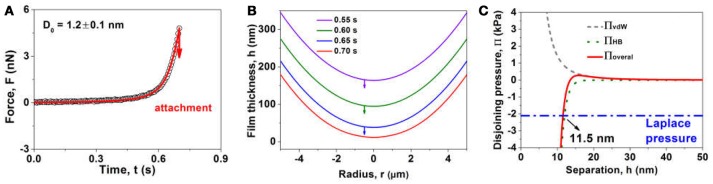
**(A)** Interaction forces (open symbols: experiment results, solid curve: theoretical calculations), **(B)** calculated thin water film profiles at different times, and **(C)** calculated disjoining pressure profiles due to vdW, hydrophobic (HB) and their overall interactions, between an air bubble (*R* = 69 μm) and a MoS_2_ basal plane in 500 mM NaCl at *v* = 1 μm/s.

Figure 8A shows the interaction between air bubble and MoS_2_ basal plane in 500 mM NaCl at *v* = 1 μm/s. The measured force profile (open symbols) shows a sudden “jump-in” behavior at some critical separation during approach, revealing that the bubble was attached to the basal plane, which was also confirmed by the optical microscope. It is noted that the EDL interaction is significantly suppressed in 500 mM NaCl and the vdW interaction is repulsive at any separation for the air-water-MoS_2_ system (Xie et al., 2017d). Thus, the observed bubble attachment must be induced by the attractive hydrophobic interaction that has been incorporated into the aforementioned theoretical model. The fitted results showed the decay length of hydrophobic interaction *D*_0_ = 1.2 ± 0.1 nm, which was the same as our previously reported *D*_0_ value at lower salinity (i.e., 1 and 100 mM NaCl) (Xie et al., 2017d), suggesting that the salt concentration plays a negligible role in the hydrophobic effect here. The calculated thin water film profiles at different times in Figure 8B shows that the bubble gradually approached the basal plane until the attachment occurred at the critical central separation of 11.5 nm (the red curve at 0.70 s), where the overall disjoining pressure just exceeded the Laplace pressure of the bubble (Figure 8C) and a pimple was formed at the central portion of bubble surface. The calculated disjoining pressure profiles in Figure 8C also indicate the hydrophobic attraction is much stronger than the vdW repulsion, which is the driving force for the bubble attachment on MoS_2_ basal plane.

**Figure 9:**
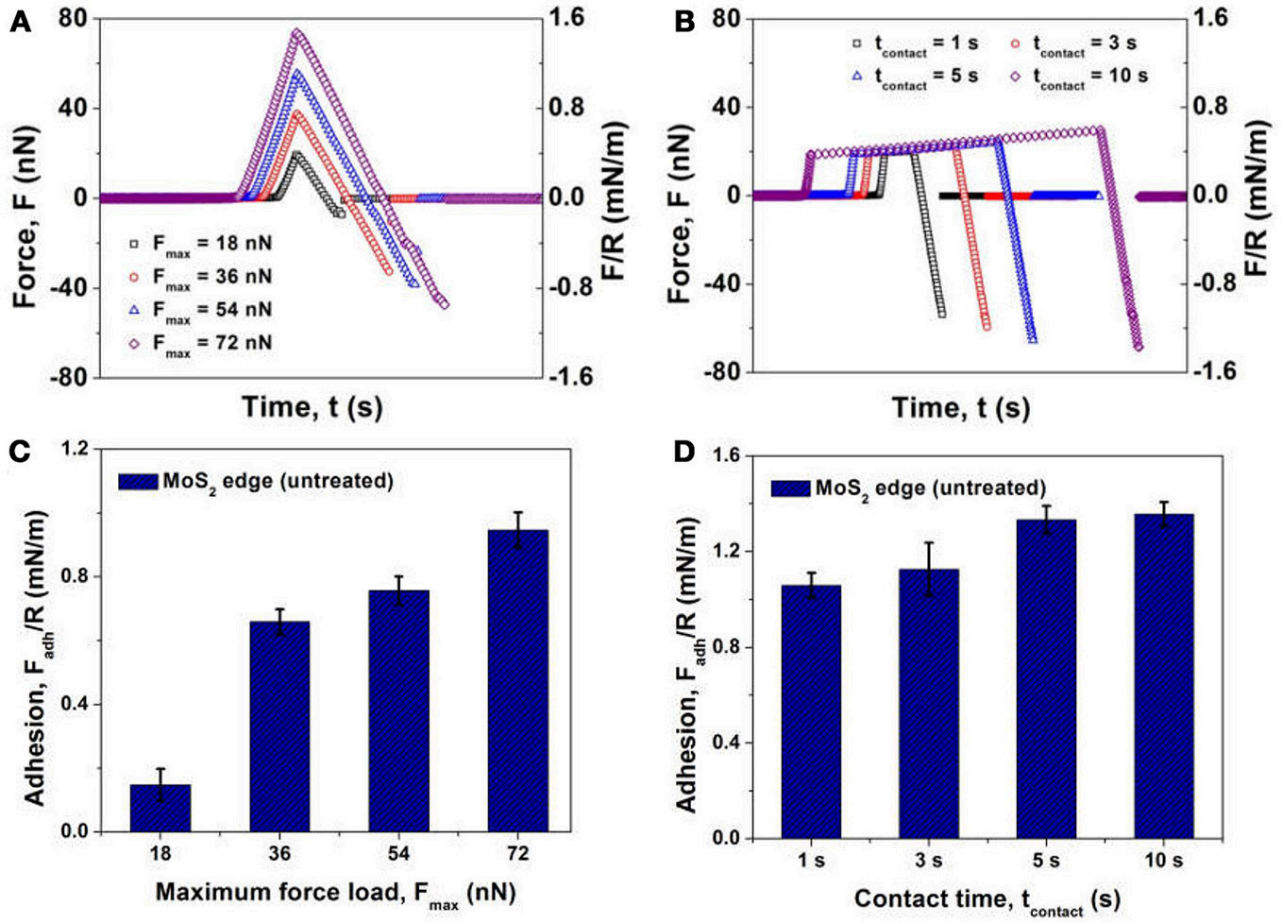
Interaction force *F* and normalized interaction *F/R* between an air bubble (*R* = 50 μm) and an untreated MoS_2_ edge plane in 500 mM NaCl at *v* = 1 μm/s under the influence of **(A)** maximum force load *F*_*max*_ and **(B)** contact time *t*_*contact*_ at a *F*_*max*_ ~18 nN. Normalized interfacial adhesion *F*_*adh*_*/R* measured between an air bubble and an untreated MoS_2_ edge plane in 500 mM NaCl at *v* = 1 μm/s under the influence of **(C)** maximum force load *F*_*max*_ and **(D)** contact time *t*_*contact*_ at a *F*_*max*_ ~18 nN.

The interaction forces measured between air bubble and MoS_2_ edge plane in 500 mM NaCl at *v* = 1 μm/s under the influence of maximum force load *F*_*max*_ and contact time *t*_*contact*_ (under *F*_*max*_) are shown in Figure 9. Evidently no “jump-in” behavior was observed during the approach-retraction cycle at *F*_*max*_ = 18–72 nN and *t*_*contact*_ = 0–10 s. The measured force results could not be fully interpreted by the aforementioned theoretical model (equations 1 and 2), indicating that other parameters, such as surface roughness of the edge plane due to polishing defects and aggregates, had an influence on the bubble-surface interaction. When the cantilever-anchored bubble approached the edge plane, a strong repulsion was detected. During retraction of the cantilever, the repulsion gradually decreased and a strong adhesion was detected, which was most likely due to the interaction of the hydrophobic domains (i.e., polishing defects/aggregates) of MoS_2_ edge plane with the bubble surface in contact during approach. With *F*_*max*_ increasing from 18 nN to 36, 54, and 72 nN in Figures 9A,C, the measured adhesion increased from 0.14 mN/m to 0.65, 0.76 and 0.95 mN/m due to the enlarged contact area between the bubble surface and the edge plane. When the bubble probe approached the edge plane and remained in contact for *t*_*contact*_ = 1 s at *F*_*max*_ ~18 nN (Figures 9B,D), the adhesion measured significantly increased to 1.08 mN/m since the polishing defects and aggregates on the edge plane could have more time to interact with the bubble surface. As shown in Figures 9B,D, continuously increasing *t*_*contact*_ to 3, 5 and 10 s only resulted in the slight rise of adhesion to 1.19, 1.31, and 1.37 mN/m, respectively, revealing that a few seconds of contact could be sufficient to ensure strong contact and adhesion between the bubble surface and the polishing defects/aggregates on MoS_2_ edge plane.

**Figure 10:**
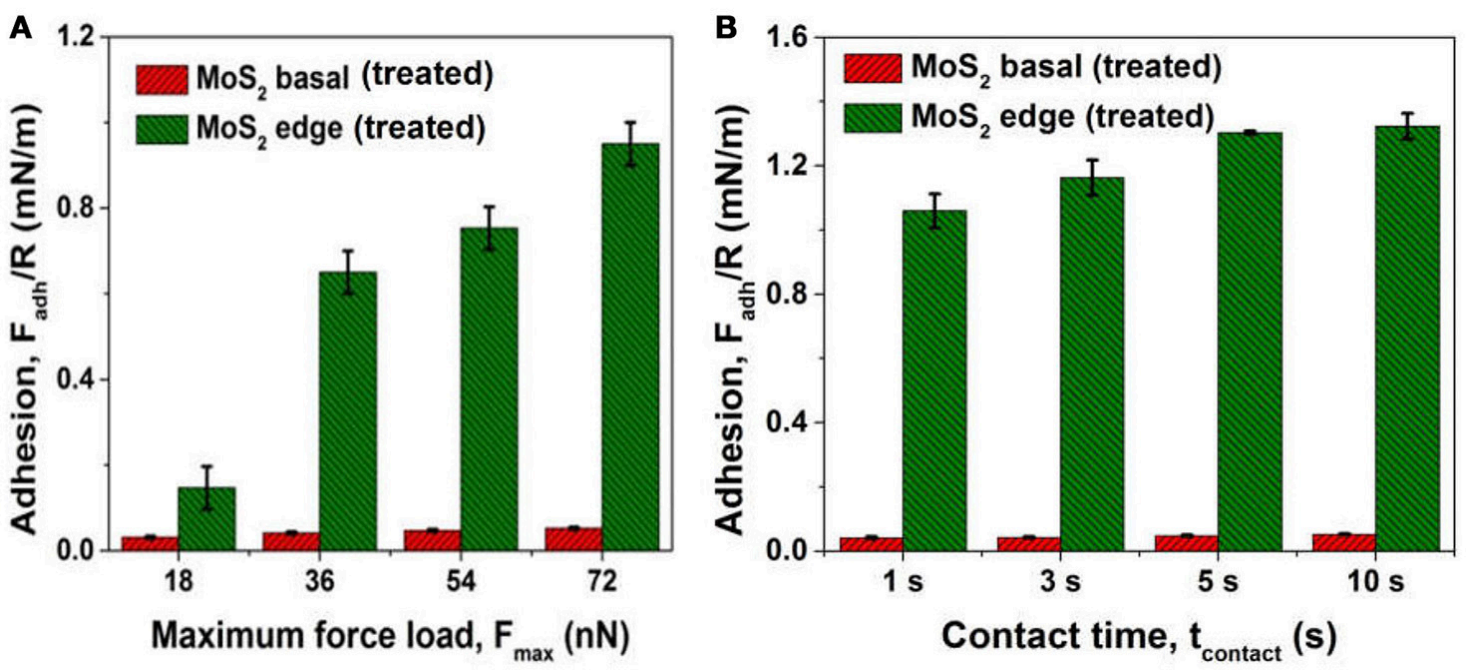
Normalized interfacial adhesion *F*_*adh*_*/R* measured between an air bubble and a treated MoS_2_ basal or edge plane conditioned in 100 ppm CMC solution in 500 mM NaCl at *v* = 1 μm/s under the influence of **(A)** maximum force load *F*_*max*_ and **(B)** contact time *t*_*contact*_ at a *F*_*max*_ ~18 nN.

After conditioned in 100 ppm CMC solution at pH 9 for 30 min, the treated MoS_2_ basal and edge planes were used for force measurements with air bubbles in 500 mM NaCl at *v* = 1 μm/s. For both the basal and edge planes, no obvious “jump in” behavior of the air bubbles was observed during the approach process, while adhesion could be detected during the surface separation. The interaction between air bubble and treated MoS_2_ basal plane is illustrated in Figure S1. The normalized interfacial adhesion *F*_*adh*_*/R* measured between air bubble and treated MoS_2_ basal or edge plane is shown in Figure 10. The treated edge plane after polymer adsorption showed similar interfacial adhesion as that without polymer adsorption (Figures 9C,D), which was attributed to the negligible adsorption of CMC on the edge plane. On the other hand, the polymer film formed on the basal plane could effectively inhibit the attraction and jump-in attachment behavior of the bubble-basal plane; while the measured interfacial adhesion during separation was about 10-fold weaker than the edge case, which was most likely due to the presence of hydrated CMC chains adsorbed on the basal plane.

Based on the bubble-MoS_2_ force results, it is revealed that the size of molybdenite particles could affect the bubble-mineral attachment and flotation performance due to different basal/edge ratios. The bubble-mineral attachment is governed by the combined influence of hydrodynamic and surface interactions (Xie et al., 2015, 2017a). The particle size can affect the hydrodynamic force, which will influence the bubble-mineral attachment. Moreover, molybdenite sheets of different sizes exhibit different basal/edge ratios, with larger basal/edge ratio and more hydrophobic properties for larger particles (Lu et al., 2015). The change of surface characteristics will also influence the bubble-mineral attachment. It is of both fundamental and practical importance to establish the correlation between the flotation behavior and the surface interaction mechanism at the nanoscale, which could provide useful information to better modulate the related process at macro-scale.

## Conclusions

In this work, AFM imaging and force measurements were employed to directly probe the adsorption of a polymer (i.e., CMC) on both MoS_2_ basal and edge surfaces as well as their interaction mechanism with air bubbles. AFM imaging showed the increased polymer coverage on the basal plane with elevating polymer concentration. As compared to the formation of a compact polymer layer on the basal plane at 100 ppm CMC, the polymer adsorption on the edge plane at the same concentration was almost negligible, which coincided with water contact angle results. Direct force measurements between CMC functionalized AFM tip and MoS_2_ showed about one order of magnitude higher adhesion on the basal plane than on the edge surface, which could be attributed to their difference in surface hydrophobicity and surface charge. For the bubble-MoS_2_ interaction, it was found that the polymer treatment could significantly influence the surface forces between air bubbles and basal plane while it had almost negligible impact on the bubble-edge interaction. The adsorbed polymers on the basal plane led to a strong repulsion during the bubble approaching process, as compared to the “jump-in” behavior and bubble attachment observed for the untreated basal plane case. This study provides quantitative information on the interactions of a model polymer depressant (i.e., CMC) and MoS_2_ basal/edge surfaces as well as how the polymer treatment on MoS_2_ basal/edge surfaces influences their anisotropic interaction mechanism with air bubbles at the nanoscale. Our results have implications for the fundamental understanding of bubble-mineral-additive interaction mechanisms in froth flotation and other related interfacial processes.

## Author contributions

LX and JW conducted the experiments. LX, JW, JH, XC, and HoZ conducted the data analysis. HoZ conceived the project and supervised the research. All authors contributed to the writing of the manuscript.

### Conflict of interest statement

The authors declare that the research was conducted in the absence of any commercial or financial relationships that could be construed as a potential conflict of interest.
