# Supplementary material for: Anisotropic Polymer Adsorption on Molybdenite Basal and Edge Surfaces and Interaction Mechanism With Air Bubbles
Source: Front Chem. 2018 Aug 20;6:361. doi: 10.3389/fchem.2018.00361 (PMC6124653; doi:10.3389/fchem.2018.00361)
Supplement: Supplementary file 1 [file Image_1.pdf]

Supporting Information

Anisotropic Polymer Adsorption on Molybdenite Basal and Edge Surfaces and Interaction Mechanism with Air Bubbles

*Lei Xie[1], Jingyi Wang[1], Jun Huang[1], Xin Cui[1], Xiaogang Wang[2], Qingxia Liu[1], Hao Zhang[1], Qi Liu[1], and Hongbo Zeng[1,]**

[1]Department of Chemical and Materials Engineering, University of Alberta, Edmonton, Alberta, T6G 1H9, Canada

[2] College of Material Science & Engineering, Heavy Machinery Engineering Research Center of Education Ministry, Taiyuan University of Science and Technology, Taiyuan 030024, China

**Email: hongbo.zeng@ualberta.ca, Phone: +1-780-492-1044, Fax: +1-780-492-2881*

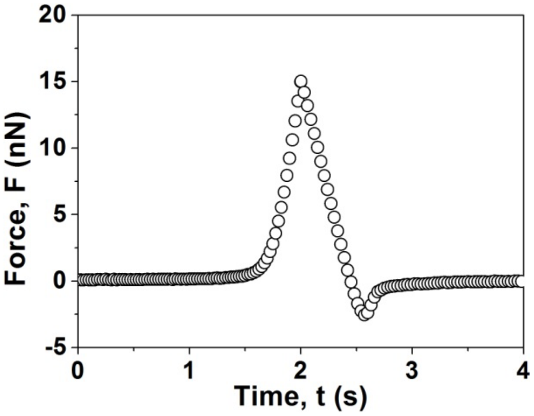

Figure S1. Interaction forces between an air bubble (R = 74 μm) and a treated MoS_2 basal plane conditioned in 100 ppm CMC solution in 500 mM NaCl at v = 1 μm/s.
